# A High-Strength, Anti-Swelling Sodium Alginate/Polyacrylamide Hydrogel Strain Sensor for Underwater Motion Monitoring and Information Transmission

**DOI:** 10.3390/gels12060468

**Published:** 2026-05-28

**Authors:** Xuecui Song, Jing Guo, Wei Chen, Mengya Liu, Yihang Zhang, Wenhui Xiao, Fucheng Guan

**Affiliations:** School of Textile and Material Engineering, Dalian Polytechnic University, Dalian 116034, China; songxuecui@163.com (X.S.); ivychen0310@163.com (W.C.); liumengya08@163.com (M.L.); 18263825182@163.com (Y.Z.); 15832395178@163.com (W.X.); gfc6322577@163.com (F.G.)

**Keywords:** underwater communication, wearable strain sensor, ionic conductive hydrogel, anti-swelling

## Abstract

Recently, conductive hydrogels have gained extensive applications in flexible wearable electronics and have garnered considerable attention. However, their inherent swelling behaviour and limited mechanical strength have hindered their further development. In this study, a polyacrylamide/sodium alginate (PAM/SA, PS)-based hydrogel with high mechanical strength and anti-swelling properties was prepared by combining mechanical stretching–drying pretreatment with a bimetallic ion (Li^+^/multivalent metal ion) post-soaking strategy. Among multivalent metal ions (Ca^2+^, Al^3+^, and Zr^4+^), the Al^3+^-crosslinked hydrogel (PS-Al^3+^) demonstrated outstanding overall performance. It exhibited excellent mechanical properties, with tensile strength, elongation at break, and impact strength reaching 9.71 MPa, 993.53%, and 75 MJ/m^3^, respectively. Its dense network structure also gave it excellent anti-swelling properties (swelling ratio of 14%). As a strain sensor, the PS-Al^3+^ hydrogel displayed good conductivity (1.33 S/m), high sensitivity (GF = 2.25), fast response (response time of 403 ms), and negligible hysteresis (recovery time of 407 ms). Benefiting from its exceptional resistance to expansion, the material’s sensor response signals in underwater environments are highly consistent with those in air. Furthermore, this sensor has been successfully applied to swimming motion monitoring and data transmission in underwater environments. This study proposes a novel, low-cost, and simple approach for developing flexible sensors suitable for underwater environments.

## 1. Introduction

Flexible wearable sensors show great potential in applications such as activity monitoring, disease detection, and human–computer interaction [1,2,3,4,5]. Researchers have made substantial efforts to develop high-performance sensing materials [6]. Conductive hydrogels, with their flexibility, biocompatibility, high conductivity, tunability, and strong resemblance to biological systems, have made ideal materials for flexible sensors. Recent years have seen significant progress in their practical applications [7,8,9]. Unfortunately, the loose network structure and lack of energy dissipation mechanisms in hydrogels often result in limited mechanical strength, which greatly restricts their application scope and lifespan [10,11]. Furthermore, the inherent hydrophilicity of hydrogels leads to unavoidable excessive swelling in aquatic environments, which significantly hinders their use underwater [12]. This is because swelling disrupts the internal network structure of hydrogels, adversely affecting their mechanical properties and conductivity, consequently leading to deterioration or even loss of sensing performance [13,14]. Thus, the creation of hydrogels that integrate excellent mechanical properties, swelling resistance, and consistent underwater sensing capabilities will propel the advancement of flexible sensors.

To date, the scientific literature has reported two effective strategies for designing anti-swelling hydrogels [15]. Among these, the introduction of hydrophobic components serves as a direct method to inhibit swelling by reducing the material’s overall affinity for water [16,17,18,19]. However, this approach introduces secondary complications, as hydrophobic elements typically resist integration with aqueous solvents, often necessitating the use of surfactants [20,21]. Moreover, the non-conductive nature of these hydrophobic additions can inadvertently diminish the hydrogel’s overall electrical performance [22]. An alternative strategy involves the design of sophisticated multi-crosslinking networks [23,24,25]. The synergistic effects resulting from various physical and chemical interactions can increase the crosslinking density of the hydrogel, enhance the elastic recovery of its network (i.e., resistance to swelling), and simultaneously limit the extent of polymer chain extension and the deformability of the network structure, thereby markedly improving its resistance to swelling [26,27]. Furthermore, hydrogels prepared using a multi-crosslinking strategy exhibit tunable mechanical strength, which is effectively regulated by controlling the crosslinking density [28]. However, anti-swelling hydrogels with dense network structures often show reduced flexibility. Furthermore, a dense network structure can restrict ion migration, leading to decreased conductivity [29,30,31]. Therefore, judiciously selecting mutually complementary interactions to ensure the hydrogel exhibits outstanding anti-swelling properties while synergistically enhancing its strength, toughness, and fatigue resistance and maintaining ideal electrical conductivity remains a major challenge.

Polyacrylamide/sodium alginate (PAM/SA) dual-network hydrogels are a widely used class of hydrogel systems. Among these, SA is exceptionally well-suited for structural regulation given its abundance, high functional group content, and favorable biocompatibility [32,33]. Nevertheless, the network structure constructed from this component is relatively loose, resulting in poor overall mechanical strength of the hydrogel. Thus, traditional PAM/SA network structures require optimization [34]. To date, incorporating polyvalent metal ions into hydrogels is a common approach for strengthening such hydrogels [35,36,37,38]. Nevertheless, relying solely on the formation of coordination bonds provides only a limited improvement in mechanical properties. Furthermore, enhancements in fatigue resistance, swelling resistance, and freeze resistance remain insufficient, making it difficult to fulfill the required mechanical properties and environmental adaptability [39].

Traditional ion-conducting hydrogels often introduce ion crosslinking through direct monometallic ion immersion to improve mechanical properties and conductivity [40,41,42]. However, the prepared ion-conductive hydrogels often struggle to balance mechanical properties and electrical conductivity, and their anti-swelling properties are also unsatisfactory. To address this, this work proposes a simple strategy: adding mechanical pre-stretching and pre-drying processes before ion immersion to induce molecular chain orientation and network densification. Simultaneously, to ensure the hydrogel’s conductivity, a bimetallic ion immersion strategy combining multivalent and monovalent ions is employed. The highly conductive monovalent Li^+^ ions effectively guarantee the conductivity required for hydrogel sensing. Based on this, this work designs a mechanical pre-stretching, pre-drying, and bimetallic ion coordination crosslinking strategy, successfully preparing an ion-conducting hydrogel with multiple interactions, high strength, and anti-swelling properties. Specifically, the initial hydrogel (PAM/SA, PS) was first pre-stretched and dried to form a dense and ordered primary network structure (covalently cross-linked network). Then, a secondary network structure was formed by utilizing the strong coordination between multivalent metal ions (Ca^2+^, Al^3+^, Zr^4+^) and carboxyl groups. This dense dual-network structure enhances the hydrogel’s network density, energy dissipation capacity, and environmental stability. Among various valence states, the Al^3+^ cross-linked hydrogel (PS-Al^3+^) exhibited superior mechanical properties and excellent anti-swelling ability. Thanks to its excellent swelling characteristics, this hydrogel exhibited stable conductivity and sensing capabilities in underwater environments. Sensors based on PS-Al^3+^ hydrogels can monitor swimming postures and underwater communications in aquatic environments. Furthermore, compared with previously reported conductive hydrogels, PS-Al^3+^ hydrogel exhibit superior overall performance (Table 1), making it a highly promising candidate material for flexible wearable sensors.

## 2. Results and Discussion

### 2.1. Design Strategies and Structural Characterization of Anti-Swelling Hydrogels

**Figure 1:**
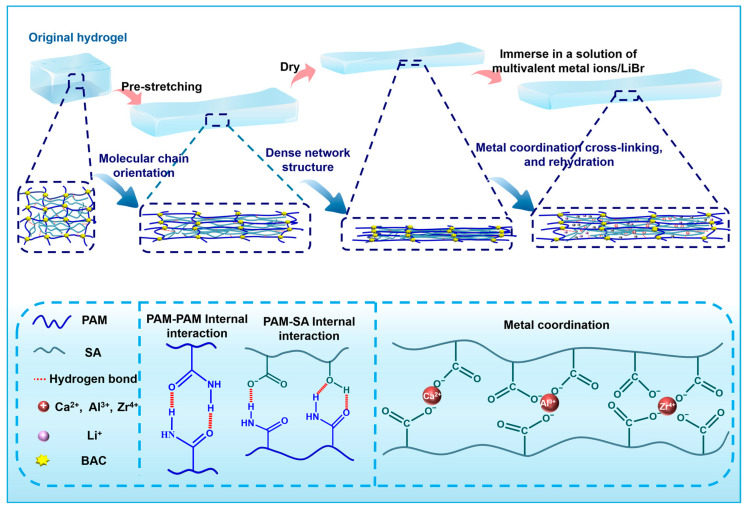
The preparation process and mechanism of anti-swelling hydrogels.

This study proposes a simple method that combines mechanical stretching with a drying process and a post-soaking strategy using bimetallic ions (Li^+^/multivalent metal ions) to prepare robust hydrogels suitable for underwater sensing (Figure 1). A chemically cross-linked PAM/SA (PS) hydrogel was used as the matrix. First, the initial hydrogel (PS) was mechanically stretched to orient and densify the molecular chains. The hydrogel was then dried to induce network shrinkage, thereby forming a dense, ordered polymer network structure (primary network). Lastly, the desiccated PS hydrogel was soaked in a composite salt solution containing multivalent metal salts (including Ca^2+^, Al^3+^, Zr^4+^) and a high concentration of LiBr (50 wt%) to undergo metal coordination and rehydration, thereby forming a secondary network. Ultimately, a dense polymer network with strong metal-coordinating cross-links was obtained. The primary rationale for selecting a metal salt solution is that multivalent metal cations can form metal-coordinating bonds with the –COO^−^ groups on the sodium alginate chains. This process not only introduces energy dissipation mechanisms into the hydrogel network to enhance mechanical properties, but also increases the network density, thereby helping to improve resistance to swelling. This coordination capability can be quantitatively evaluated by the ionic potential (φ = Z/r, where Z is the ion valence and r is the ionic radius), a parameter that reflects the charge density and coordination ability of the ions [48,49,50]. Consequently, the crosslinking density and microstructure of the polymer network can be tuned by varying the valence state and type of metal ions. Furthermore, the incorporation of high concentrations of LiBr enhances the hydrogel’s electrical conductivity and, through strong ion–dipole interactions, confers freeze resistance to the hydrogel. Through the design strategies, we prepared a series of ion-conductive hydrogels with tunable mechanical performance, electrical conductivity, and anti-swelling properties to meet the diverse requirements of flexible sensing in various applications.

**Figure 2:**
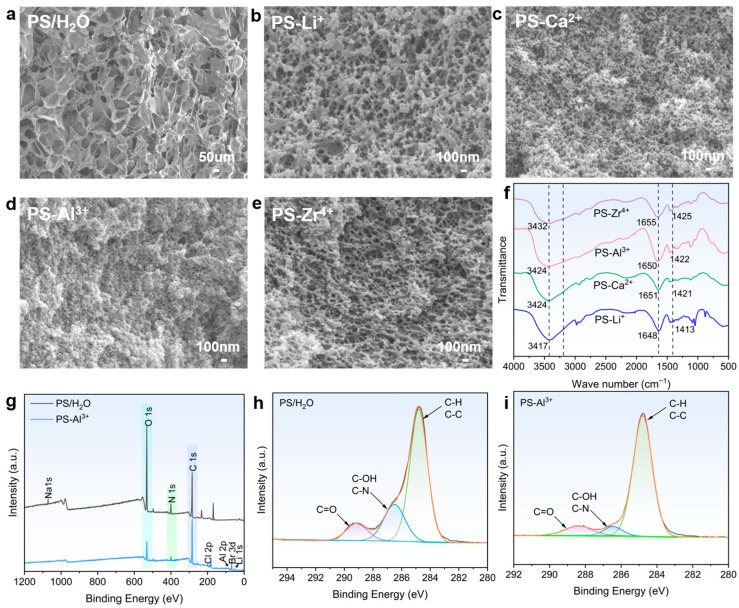
SEM images of (**a**) PS/H_2_O, (**b**) PS-Li^+^, (**c**) PS-Ca^2+^, (**d**) PS-Al^3+^, and (**e**) PS-Zr^4+^ hydrogels. (**f**) FTIR spectra of PS-Li^+^, PS-Ca^2+^, PS-Al^3+^, and PS-Zr^4+^ hydrogels. (**g**) Full XPS spectra of the PS/H_2_O and PS-Al^3+^ hydrogels. (**h**) C 1s narrow spectrum of PS/H_2_O hydrogel; (**i**) C 1s narrow spectrum of PS-Al^3+^ hydrogel.

The morphology of the hydrogels was characterized using scanning electron microscopy (SEM) (Figure 2a–e). Observations show that all hydrogels have a porous structure, which provides good transport channels for ion migration. Among them, PS/H_2_O hydrogels exhibit a loose macroporous structure due to their sparse polymer network crosslinking (Figure 2a). In contrast, the ionically crosslinked hydrogels exhibit a denser network structure and a significantly reduced pore size, indicating that ion crosslinking effectively promotes the densification of the polymer network (Figure 2b–e). Among them, the PS-Al^3+^ hydrogel exhibits the densest pore structure (Figure 2d), which is attributed to the strong coordination ability of Al^3+^, enabling it to form a stable ternary cross-linked structure with the carboxyl groups on the SA chains, thereby constructing a network with higher cross-linking density. Intermolecular interactions within the hydrogel network were further investigated using Fourier-transform infrared spectroscopy (FTIR). Figure S1 (Supplementary Materials) shows that the peaks of SA at 3432 cm^−1^, 1032 cm^−1^, 1620 cm^−1^, and 1416 cm^−1^ correspond to the O–H stretching vibration, the C–O (C–O–C) symmetric stretching vibration, and the asymmetric and symmetric stretching vibrations of the –COO^−^ group, respectively [51]. The PS/H_2_O hydrogel spectrum includes characteristic peaks of SA, with a typical peak at 3447 cm^−1^ (O–H stretching). Peaks at 3185 cm^−1^ (N–H stretching), 1655 cm^−1^ (–C=O), and 1610 cm^−1^ (amide II band, N–H bending) originate from the amide groups of PAM [52]. These results indicate that the hydrogel was successfully prepared [53]. Compared to pure SA, the O–H stretching peak of the PS/H_2_O hydrogel exhibited a blue shift and enhanced absorption, suggesting the formation of hydrogen bonds between the SA and PAM chains [54,55]. In hydrogels cross-linked by polyvalent metal ions, the asymmetric stretching vibration peak of the carboxyl group at 1620 cm^−1^ shifted to the left, indicating the formation of metal-coordinate bonds between –COO^−^ and the metal ions (Figure 2f) [56]. Furthermore, X-ray photoelectron spectroscopy (XPS) was further employed to characterize the chemical structures of the hydrogels. As shown in Figure 2g, the pristine PS/H_2_O hydrogel mainly contained four elements: C, N, O, and Na. In contrast, the PS-Al^3+^ hydrogel exhibited new peaks corresponding to Br, Li, Al, and Cl, indicating that Li^+^ and Al^3+^ ions were successfully introduced into the hydrogel network. The corresponding high-resolution XPS spectra are presented in Figure S2 (Supporting Information). As shown in Figure 2h, the high-resolution C 1s narrow spectrum of the PS/H_2_O hydrogel exhibits characteristic peaks at 284.8 eV, 286.5 eV, and 289.1 eV, attributed to C–C/C–H, C–O/C–N, and C=O, respectively. After the introduction of the bimetallic salts (Li^+^ and Al^3+^), the positions of the C–OH/C–N and C=O peaks in the C 1s spectrum of the PS-Al^3+^ hydrogel shifted to 286.45 eV and 288.38 eV, respectively, confirming the formation of intermolecular hydrogen bonds as well as lithium bonds promoted by metal coordination bonds and electrostatic interactions [57]. In addition, the elemental distribution and content of the PS-Al^3+^ hydrogel were analyzed using EDS mapping images (Figure S3, Supporting Information) and EDS spectra (Figure S4, Supporting Information). Notably, elements such as C, N, O, Br, Al, and Cl were uniformly distributed throughout the hydrogel. This uniform distribution clearly indicates that AlCl_3_ and LiBr have been successfully incorporated into the PS hydrogel system.

### 2.2. Mechanical Properties

**Figure 3:**
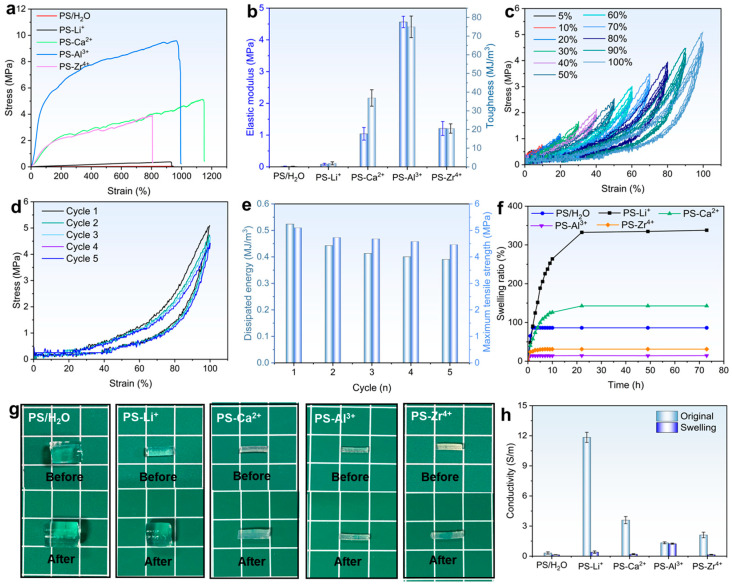
(**a**) Tensile stress–strain curves of PS/H_2_O, PS-Li^+^, PS-Ca^2+^, PS-Al^3+^, and PS-Zr^4+^ hydrogels, along with their corresponding (**b**) elastic moduli and toughness. (**c**) Cyclic tensile loading and unloading curves of the PS-Al^3+^ hydrogel under different strains. (**d**) A magnified view of the five consecutive cyclic curves at 100% strain shown in (**c**), along with the corresponding (**e**) energy dissipation and maximum tensile strength. (**f**) Swelling kinetics curves of PS/H_2_O, PS-Li^+^, PS-Ca^2+^, PS-Al^3+^, and PS-Zr^4+^ hydrogels at room temperature, along with their corresponding (**g**) comparative photographs after 72 h of swelling and (**h**) electrical conductivity.

As displayed in Figure 3a and Figure S5 (Supplementary Information), the tensile strength of the ionically cross-linked hydrogel was significantly enhanced compared to the original PS/H_2_O hydrogel. In particular, the tensile strength of the PS-Al^3+^ hydrogel reached as high as 9.71 MPa, representing a 193-fold increase compared to PS/H_2_O. In addition, the stress statistics shown in Figure S5 (Supplementary Materials) indicate that the tensile strengths of PS-Li^+^, PS-Ca^2+^, and PS-Al^3+^ reach 0.62 MPa, 4.58 MPa, and 9.71 MPa, respectively. This suggests that the tensile strength of the hydrogels increases with the valence of the metal ions. This phenomenon stems from stronger metal-coordination interactions between high-valent metal ions and polymer chains, which enhance interchain interactions [48,49]. Furthermore, the improvement in the mechanical properties of the hydrogel is also related to a salt-induced toughening mechanism. Specifically, during the bimetallic ion soaking process, the high concentration of LiBr screens electrostatic repulsion, promotes interchain aggregation, enhances intermolecular interactions, and improves mechanical properties. Moreover, as shown in Figure S5 (Supplementary Materials), the fracture elongation of hydrogels cross-linked with multivalent metal ions (Ca^2+^, Al^3+^, and Zr^4+^) decreases as the valence of the ions increases. This is because the increased cross-linking density restricts chain mobility, leading to increased material stiffness. Nevertheless, PS-Al^3+^ still exhibited an elongation at break of up to 993.53%. This is primarily associated with the dynamic reversibility of the metal coordination bonds. Specifically, during tensile loading, the coordination bonds can dissociate and reform, thereby effectively dissipating energy, suppressing crack propagation, and maintaining the material’s ductility. However, compared to the PS-Al^3+^ hydrogel, the tensile strength (3.92 MPa) decreased significantly when the higher-valent Zr^4+^ was introduced. The reason is that Zr^4+^ has a high charge density and extremely strong hydrolytic activity. Upon entering the gel pores, it preferentially self-polymerizes to form Zr–O–Zr oxo-bridged clusters, which greatly reduces the number of active coordination sites available on the outside for binding with carboxyl groups, leading to a marked decrease in effective crosslinking density. A similar trend was also observed in terms of toughness and elastic modulus, with the PS-Al^3+^ hydrogel exhibiting a high toughness of 75 MJ/m^3^ (Figure 3b). Owing to its superior combination of mechanical properties, PS-Al^3+^ was therefore selected as the primary system for subsequent investigation.

Outstanding fatigue resistance and self-healing capabilities are also key factors enabling hydrogels to achieve long-term reusability and stable signal output. The cyclic load–unload curves and corresponding energy dissipation under different tensile strains (0–100%) are presented in Figure 3c and Figure S6 (Supplementary Materials). As stress increased, both the hysteresis loop area and dissipated energy increased progressively. This indicates that the material is capable of effectively dissipating energy during deformation, thereby preventing structural damage or fracture [58]. In addition, to further investigate the fatigue resistance of the PS-Al^3+^ hydrogel, its continuous cyclic loading-unloading curves under the same tensile strain were examined. Taking five consecutive cycles at 100% strain in Figure 3c as an example, its local magnified view and the corresponding energy dissipation are shown in Figure 3d and Figure 3e, respectively. As shown in Figure 3d,e, a distinct hysteresis loop was observed during the first cycle, with an energy dissipation of approximately 0.52 MJ/m^3^. The initial energy loss stems from irreversible bond breakage within the hydrogel network during the first tensile cycle. These bonds act as sacrificial bonds and cannot be fully restored in subsequent cycles [59]. However, during cycles 2 through 5, the load–unload curves coincided nearly exactly, suggesting that the PS-Al^3+^ hydrogel exhibited outstanding stability and fatigue resistance. These results strongly demonstrate that incorporating multiple physical and chemical interactions into the hydrogel network enables effective improvement of its mechanical properties [60].

### 2.3. Anti-Swelling Properties

The swelling behavior of hydrogels compromises the integrity of their internal network, leading to degraded mechanical and electrical properties as well as distorted sensing signals, which severely limit their practical applications in underwater environments [15,61]. We immersed PS/H_2_O, PS-Li^+^, PS-Ca^2+^, PS-Al^3+^, and PS-Zr^4+^ hydrogels in deionized water for 72 h to study their swelling kinetics. The swelling profiles and corresponding digital photographs of these hydrogels before and after 72 h of immersion are presented in Figure 3f and Figure 3g, respectively. Clearly, the PS/H_2_O hydrogel without ionic crosslinking swelled rapidly within the first two hours, reaching an equilibrium swelling ratio of 86%. This means that water molecules can easily traverse the PS/H_2_O hydrogel’s network boundaries [62,63]. Notably, the PS/H_2_O hydrogel underwent a three-hour rehydration process during preparation, resulting in a certain degree of pre-swelling; this explains the relatively low swelling ratio observed in the subsequent swelling kinetics tests. Observation of Figure 3f,g reveal that PS-Li^+^ exhibits an exceptionally high swelling rate (up to 338%), accompanied by a dramatic expansion in hydrogel volume. This is primarily attributed to the significant osmotic pressure difference between the interior and exterior of the hydrogel, which drives a large number of water molecules to permeate into the network (Donnan effect). In addition, the large hydration radius of Li^+^ effectively increases the spacing between polymer chains. At the same time, high concentrations of salt ions weaken the interactions between polymer chains, reducing the density of the network structure. Moreover, the weak coordination of Li^+^ makes it difficult to construct a rigid and compact crosslinked structure, ultimately resulting in more significant swelling behavior. In contrast, in hydrogels crosslinked by multivalent metal ions (Ca^2+^, Al^3+^, Zr^4+^), the network structure is restricted by the crosslinking and cannot accommodate such a large amount of water, thus exhibiting a lower swelling ratio. Among these, the PS-Al^3+^ hydrogel, which possesses a highly dense network structure, exhibits significantly superior resistance to swelling compared to the PS-Ca^2+^ and PS-Zr^4+^ hydrogels, with a swelling rate as low as 14% and negligible volume change. These results further confirm that a dense network structure can effectively prevent water penetration and inhibit swelling [18,61,64]. Moreover, this structural stability directly influences the material’s electrochemical performance over time. When examining the conductivity of PS/H_2_O, PS-Li^+^, PS-Ca^2+^, PS-Al^3+^, and PS-Zr^4+^ hydrogels following a 72 h immersion in deionized water (Figure 3h), the benefits of the PS-Al^3+^ network become even more apparent. Because the PS-Al^3+^ hydrogel effectively resists swelling, its conductivity remained largely stable. However, the electrical conductivity of the other four hydrogels decreased significantly upon swelling, which is attributed to the migration of metal ions out of the hydrogel network during the swelling process [42]. In addition, the swelling behavior of the PS-Al^3+^ hydrogel in different solutions (artificial seawater and 0.5 M NaCl solution) was further investigated to examine the general applicability of its anti-swelling property. As shown in Figure S7, the equilibrium swelling ratios of the PS-Al^3+^ hydrogel in artificial seawater and 0.5 M NaCl solution were −7.8% and −9.8%, respectively. This can be attributed to the Hofmeister salting-out effect, which causes dehydration and aggregation of polymer chains, leading to network collapse. The swelling performance in seawater is also related to the additional ionic crosslinking between ions such as Mg^2+^ and Ca^2+^ in seawater and the carboxyl groups in SA. These results indicate that the PS-Al^3+^ hydrogel exhibits excellent anti-swelling performance in different solutions, which greatly broadens its application range. Furthermore, comparing the conductivity before and after swelling, it was found that after reaching swelling equilibrium in artificial seawater and 0.5 M NaCl solution, the conductivity increased from an initial value of 1.33 S/m to 2.39 S/m and 2.81 S/m, respectively (Figure S8). The increase in electrical conductivity is attributed to the influx of free ions from seawater and NaCl solutions into the hydrogel network. Additionally, hydrogels used in underwater applications also require good mechanical properties. As can be seen from Figure S9, PS-Al^3+^ hydrogel exhibits good mechanical properties in seawater and 0.5 M NaCl solution due to its excellent anti-swelling properties.

### 2.4. Sensing Performance

**Figure 4:**
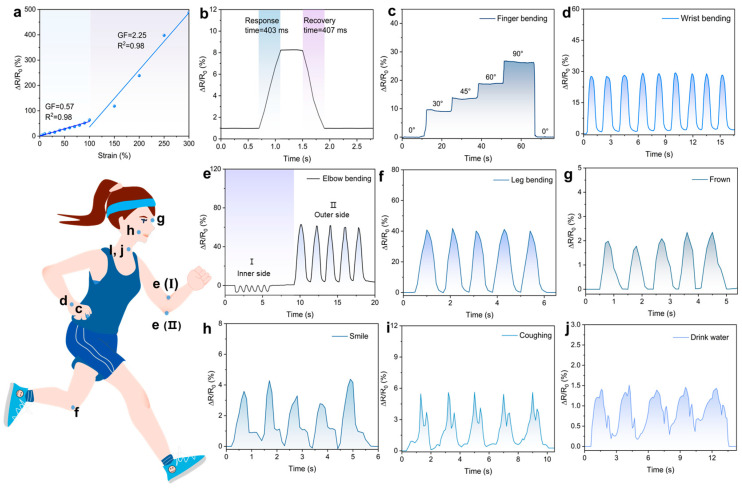
(**a**) Change in ∆*R/R*_0_ of the PS-Al^3+^ hydrogel under different strains. (**b**) Response and recovery times. Changes in ∆*R/R*_0_ of the sensor while monitoring (**c**) finger bending, (**d**) wrist bending, (**e**) elbow bending, (**f**) knee bending, (**g**) frowning, (**h**) smiling, (**i**) coughing, and (**j**) drinking.

The strain coefficient (GF) is a critical parameter for measuring the sensitivity of a strain sensor. The slope of the relative resistance change (Δ*R/R*_0_) versus strain curve is used to calculate GF. As illustrated in Figure 4a, the Δ*R/R*_0_ value of the PS-A1^3+^ hydrogel was positively correlated with the applied strain, demonstrating its strain sensitivity. The PS-Al^3+^ hydrogel exhibited two linear response regions, with GF values of 0.57 and 2.25 in the 0–100% and 100–300% ranges, respectively. This increase in sensitivity with increasing strain stems from the fact that large-scale deformation may trigger drastic changes in internal conductive pathways and ion distribution [65,66]. In addition to high strain sensitivity, hydrogel also exhibits rapid responsiveness and low hysteresis. As illustrated in Figure 4b, the sensor’s response time is 403 ms, and its recovery time is 407 ms. There is only a negligible hysteresis of 4 ms between these two processes, ensuring the possibility of real-time monitoring of various human dynamic changes. Owing to the PS-Al^3+^ hydrogel’s outstanding sensing properties, we incorporated it into a wearable strain sensor for monitoring human activity. When the sensor was attached to the finger, it accurately detected various bending angles (Figure 4c). Under a constant bending angle, the corresponding Δ*R/R*_0_ value remains constant. Furthermore, when the finger returned from a bent state to its initial straight state, ∆*R/R*_0_ rapidly returned to its initial value. The results above demonstrate the reliability and stability of hydrogel sensors in monitoring human joint movement. As shown in Figure 4d–f, when the sensor underwent repeated flexion movements at different joints, it generated stable, repeatable, and clear sensing signals. In addition, the sensor demonstrated high sensitivity in monitoring subtle physiological signals. As shown in Figure 4g,h, the sensor could accurately detect minute deformations caused by facial muscle movements, including micro expressions such as frowns and smiles. Furthermore, the sensor mounted on the throat could accurately identify and distinguish between the faint vibrations caused by two different physiological activities: coughing and drinking (Figure 4i,j). The above results indicate that this sensor holds great promise for use in wearable electronic devices.

### 2.5. Underwater Sensing Performance

**Figure 5:**
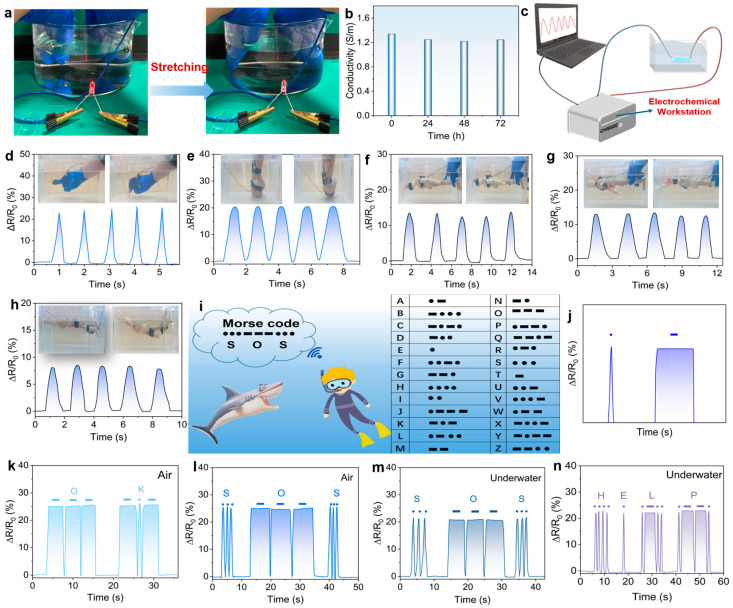
(**a**) Variation in LED brightness upon stretching the PS-Al^3+^ hydrogel in deionized water. (**b**) Conductivity of PS-Al^3+^ hydrogels in deionized water after different immersion times. (**c**) Schematic diagram of underwater sensor performance testing. Real-time underwater sensing applications: representative demonstrations of (**d**) finger bending and (**e**) wrist bending. Sensors mounted on the mannequin monitor typical swimming postures, including breathing (**f**), gliding (**g**), and kicking (**h**). Underwater communication applications: (**i**) Schematic diagram of an underwater communication system based on Morse code. (**j**) Definition of the “dot” (·) and “dash” (—) signals. Transmitting information via Morse code in different environments. In the air: (**k**) “OK”, (**l**) “SOS”. Underwater: (**m**) “SOS”. (**n**) “HELP”.

Given the PS-Al^3+^ hydrogel’s exceptional resistance to swelling and high sensitivity, we further investigated its underwater sensing performance. In underwater environments, the stability of electrical conductivity and the reliability of sensing performance determine the suitability of underwater sensors [67]. To verify the conductivity and strain sensitivity of the PS-Al^3+^ hydrogel in an underwater environment, we immersed the hydrogel in water and evaluated its conductivity and strain sensitivity by observing changes in the brightness of an LED connected in series with the hydrogel. As shown in Figure 5a, the PS-Al^3+^ hydrogel was still able to illuminate the LED underwater, and the LED brightness gradually decreased as the hydrogel was stretched. This clearly demonstrates that the PS-Al^3+^ hydrogel has good conductivity and strain sensitivity even in underwater environments. More importantly, the PS-Al^3+^ hydrogel exhibits outstanding electrical stability in an underwater environment. Its conductivity dropped only slightly from 1.33 S/m to 1.25 S/m during the first 24 h and remained stable over the subsequent 48 h (Figure 5b). This excellent conductive stability lays a reliable foundation for underwater monitoring. To investigate its practical sensing capabilities in underwater environments, the hydrogel was mounted on a finger and a wrist. As shown in Figure 5d,e, during periodic flexion and extension movements of the finger and wrist underwater, the sensor generated stable and repeatable response signals, demonstrating its application potential for monitoring human underwater movements. To further expand its application scope, the sensor was integrated into a human model to monitor various swimming strokes. Figure 5f–h show that sensors mounted on the neck, shoulders, and knees can distinguish between different swimming strokes, like breathing, arm strokes, and leg kicks. In addition to motion monitoring, this hydrogel sensor can also be used for underwater communication. Underwater communication technology is essential for protecting divers’ safety and enabling real-time data transmission. Morse code is a simple and efficient communication method that encodes and transmits information through combinations of short “dots” (·) and long “dashes” (—) (Figure 5i). When the hydrogel was installed on the index finger, its resistance changed in real time with the speed at which the finger bent, converting the finger’s movements into Morse code. We defined the transient spikes generated by rapid finger bending as “·” and the plateau-like signals generated by slow bending as “—” (Figure 5j). Figure 5k,l show volunteers successfully sending distress signals such as “OK” and “SOS” in an air environment, confirming the feasibility of converting electrical signals into Morse code. Furthermore, the sensor can stably transmit various messages—ranging from simple (e.g., “SOS”) to complex (e.g., “HELP”)—in underwater environments as well (Figure 5m,n). More importantly, the “SOS” signals generated in the air (Figure 5l) and underwater (Figure 5m) show a high degree of consistency, demonstrating the sensor’s exceptional stability and reliability in different environments. The freeze resistance of the PS-Al^3+^ hydrogel was evaluated through freeze–thaw testing and differential scanning calorimetry (DSC) analysis. After being stored at –80 °C for 2 h, the PS-Al^3+^ hydrogel retained excellent flexibility and good electrical conductivity (Figure S7, Supplementary Information), demonstrating its suitability for use in low-temperature underwater environments. In summary, the PS-Al^3+^ hydrogel holds great promise for applications such as professional swimming training, underwater communication, rescue operations, and safety alerts.

## 3. Conclusions

In this work, mechanical stretching and drying pretreatment were employed to induce molecular chain orientation and densification of the network structure in PAM/SA (PS) hydrogels. Subsequently, polyvalent metal ion salts of different valences and lithium bromide were introduced to construct an anti-swelling ionically conductive hydrogel that combines high strength and high toughness through metal coordination and electrostatic interactions. Among these, lithium bromide ensures the conductivity required for hydrogel sensing. Compared with Li^+^, Ca^2+^, and Zr^4+^, Al^3+^ exhibited excellent coordination ability, forming a denser network structure. The PS-Al^3+^ hydrogel exhibited high strength, high toughness, and excellent fatigue resistance, attributed to the synergistic interplay of a dense network structure and multiple energy dissipation mechanisms (hydrogen bonds, coordination bonds, and electrostatic interactions). This dense network structure also conferred excellent anti-swelling performance to the PS-Al^3+^ hydrogel. As a strain sensor, it can sensitively monitor human movements at various scales, such as large-scale joint movements, subtle facial expressions, and physiological signals including coughing. Thanks to its excellent resistance to swelling, the PS-Al^3+^ hydrogel was able to produce stable sensing signals in an aqueous environment that were consistent with those in air. Furthermore, in aquatic environments, this hydrogel sensor was not only capable of monitoring swimming strokes but could also use Morse code to accurately transmit various distress signals (e.g., OK, SOS, and HELP). This study provides new insights for the design and development of next-generation underwater wearable sensors and communication devices.

## 4. Materials and Methods

### 4.1. Materials

Sodium alginate (SA) was provided by Mingyue Algae Group (Qingdao, China). Acrylamide (AM, 99%), ammonium persulfate (APS), 2-hydroxy-4′-(2-hydroxyethoxy)-2-methylpropylphenyl ketone (Irgacure 2959), and aluminum chloride hexahydrate (AlCl_3_·6H_2_O, 97%) were purchased from Aladdin. N, N, N’, N’-tetramethylenediamine (TEMED, 99%), N, N’-bis(acryloyl)cystamine (BAC, 98%), lithium bromide (LiBr, 98%), and zirconyl chloride octahydrate (ZrOCl_2_·8H_2_O) were supplied by Macklin (Shanghai, China). Anhydrous calcium chloride (CaCl_2_, AR, 96%) was purchased from Tianjin Kemei Chemical Reagent Co., Ltd. (Tianjin, China).

### 4.2. Preparation of Hydrogel

The monomer AM (16 g), crosslinking agent BAC (0.048 g), photoinitiator Irgacure 2959 (0.096 g), and redox initiators APS (0.0268 g) and TEMED (40 μL) were added sequentially to the uniformly dispersed SA solution. The mixture was stirred under a nitrogen atmosphere until homogeneous. After degassing, immediately pour the solution into the mold and expose it to ultraviolet light at 365 nm for 240 min to form a polyacrylamide (PAM) network. Subsequently, the sample was subjected to heat treatment in a vacuum oven at 65°C to ensure the complete conversion of residual monomers, thereby yielding the PAM/SA (PS) composite hydrogel. The hydrogel was stretched to 300% of its original length and left at room temperature overnight to dry. Lastly, the dried sample was soaked in a mixed solution containing 50 wt% LiBr and 0.5 mol/L of various metal salts (CaCl_2_, AlCl_3_·6H_2_O, or ZrOCl_2_·8H_2_O) for 3 h. A series of ion-conductive hydrogels was thus prepared, designated as PS-Ca^2+^, PS-Al^3+^, and PS-Zr^4+^, respectively. Following the same procedure described above, dried PS hydrogels—stretched to 300% strain—were immersed in deionized water and a LiBr solution for 3 h, respectively, to prepare the control PS/H_2_O and PS-Li^+^ hydrogels.

### 4.3. Characterization

#### 4.3.1. Structural Characterization

Fourier-transform infrared (FTIR) spectra were acquired using a Spectra Two spectrometer (PerkinElmer, Waltham, MA, USA) with a scan range of 500–4000 cm^−1^. The microstructure of the hydrogels was examined by scanning electron microscopy (SEM, JSM-7800F, JEOL Ltd., Tokyo, Japan). In conjunction with SEM, an X-ray spectrometer (EDS, X-Max50, Oxford Instruments, Oxford, UK) was used to analyze the elemental distribution, composition, and concentration of the dried hydrogel. X-ray photoelectron spectroscopy (XPS, K-Alpha, Thermo Fisher Scientific, Waltham, MA, USA) was used to characterize the elemental composition and chemical state of the hydrogel. The spectra were acquired in the energy range of 0–1350 eV.

#### 4.3.2. Mechanical Testing

Tensile tests were performed using an electronic universal testing machine (CMT4503, Shandong Wanchen Testing Machine Co., Ltd., Jinan, Shandong, China) at a crosshead speed of 50 mm/min. The elastic modulus was determined from the slope of the initial linear region of the stress–strain curve. Toughness was calculated by integrating the area under the stress–strain curve from zero strain to the fracture point. Dissipated energy was obtained by calculating the area enclosed by the closed hysteresis loop in the stress–strain curve.

#### 4.3.3. Anti-Freezing Testing

The freezing-resistance of the hydrogel fibers was evaluated by differential scanning calorimetry (DSC, Q2000, TA Instruments, New Castle, DE, USA). The temperature program consisted of cooling from 25 °C to –80 °C at 5 °C·min^−1^, followed by heating from –80 °C to 25 °C at 10 °C·min^−1^.

#### 4.3.4. Anti-Swelling Testing

Weigh the prepared hydrogel samples and record their initial mass (*m*_0_), then immerse them in different solutions (deionized water, artificial seawater, and 0.5 M NaCl solution) to study their swelling behavior. Remove the swollen samples at specific time intervals and record their mass (*m_t_*) until the hydrogel mass remains constant. The swelling ratio is calculated using the following formula:

$$\mathrm{Swelling}\;\mathrm{ratio}\;(\%)=(m_{t}-m_{0})/m_{0}\times 100\%$$

Here, *m*_0_ represents the mass of the hydrogel sample before the swelling process begins, and *m_t_* represents the mass of the sample after the swelling process ends.

#### 4.3.5. Electrical Measurements

The electrical properties of the hydrogel were evaluated using a CHI660E electrochemical workstation (Chenhua Instrument, Shanghai, China). The resistance of the samples was measured via electrochemical impedance spectroscopy (EIS). The ionic conductivity (*σ*) was then calculated using the following formula:

σ=L/(R×S)

where *L*, *S*, and *R* represent the length, cross-sectional area, and measured resistance of the sample, respectively.

#### 4.3.6. Sensing Performance Testing

The sensing performance was characterized using a CHI660E electrochemical workstation (Chenhua Instrument, Shanghai, China) in conjunction with a universal testing machine (CMT4503, Shandong Wanchen Testing Machine, Jinan, Shandong, China). The relative change in resistance (Δ*R*/*R*_0_) was calculated as follows:

$$∆R/R_{0}=\left(R-R_{0}\right)/R_{0}\times 100\%$$

where *R*_0_ and *R* are the electrical resistance of the sample at its initial state and under strain, respectively.

The gauge factor (GF) of conductive hydrogels is calculated using the following formula:

$$\mathrm{GF}=(∆R/R_{0})/\varepsilon$$

where *ε* represents the applied strain.

The assembled hydrogel sensors were attached to various body parts (fingers, wrists, elbows, knees, throat, and face) to detect human motions, with electrical signal variations recorded by a CHI660E electrochemical workstation during movement.

## Figures and Tables

**Table 1:** Comparison of the properties of PS-Al^3+^ hydrogels with those of previously reported conductive hydrogels.

Samples	Strain (%)	Stress (MPa)	Anti-Freezing (°C)	Swelling Ratio (%)	Conductivity (S/m)	GF	Reference
MAlggel	≈140	1.88 ± 0.04	/	/	0.006	>1.6	[40]
PTPAVI-Al^3+^	854	0.148	−32.34	−8.3	2.165	2.01	[41]
Gel/PSAA-Al^3+^	1050	0.25	−31.58	32	0.638	1.61	[42]
PVA/AG/AMPS/PA-LiCl	≈390	0.4445	−66.58	/	7.61	1.96	[43]
PAM/PVA/SC	719	0.4443	/	/	Yes	2.17	[44]
P(AA-MEA)-CS-Fe	1199	0.462	/	≈−8	0.326	5.25	[45]
GelMA	≈160	≈0.13	/	/	0.6	≈3.28	[46]
β-CD-g-(pAAm/pAETAc)	3000	0.11	/	/	0.29	3.74	[47]
PS-Al^3+^	993.53	9.70	<−80	14	1.33	2.25	This work

Note: “/” represents not reported.

## Data Availability

The raw data supporting the conclusions of this article will be made available by the authors upon request.

## Supplementary Materials

The following supporting information can be downloaded at: https://www.mdpi.com/article/10.3390/gels12060468/s1, Figure S1. FTIR spectra of SA and PS/H_2_O; Figure S2. High-resolution XPS spectra of different elements in the PS-Al^3+^ hydrogel; Figure S3. EDS mapping of the PS-Al^3+^ hydrogel; Figure S4. EDS spectra of the PS-Al^3+^ hydrogel; Figure S5. Tensile strength and elongation at break of five hydrogels; Figure S6. Hysteretic loss of the PS-Al^3+^ hydrogel during continuous loading and unloading cycles (with the maximum strain set at 5–100%); Figure S7. Swelling ratios of PS-Al^3+^ hydrogels in artificial seawater and 0.5 M NaCl solution; Figure S8. Conductivity of PS-Al^3+^ hydrogels before and after reaching swelling equilibrium in artificial seawater and 0.5 M NaCl solution; Figure S9. Tensile strength and elongation at break of PS-Al^3+^ hydrogels before and after reaching swelling equilibrium in artificial seawater and 0.5 M NaCl solution; Figure S10. Freeze resistance of PS/H_2_O and PS-Al^3+^ hydrogels. (a) Photographs showing the mechanical flexibility of both hydrogels at −80 °C. (b) Photographs demonstrating the electrical conductivity of both hydrogels at 25 °C and −80 °C. (c) DSC curves of both hydrogels. (d) Electrical conductivity of PS-Al^3+^ hydrogel at different temperatures.

## Abbreviations

The following abbreviations are used in this manuscript:

SA	Sodium alginate
AM	Acrylamide
APS	ammonium persulfate
Irgacure 2959	2-hydroxy-4′-(2-hydroxyethoxy)-2-methylpropylphenyl ketone
AlCl_3_·6H_2_O	Aluminum chloride hexahydrate
LiBr	lithium bromide
TEMED	N,N,N’,N’-tetramethylenediamine
ZrOCl_2_·8H_2_O	Zirconyl chloride octahydrate
BAC	N,N’-bis(acryloyl)cystamine
CaCl_2_	Anhydrous calcium chloride
BACA	Linear dichroism
PS	Polyacrylamide/Sodium alginate
PS-Li^+^	Polyacrylamide/Sodium alginate-LiBr
PS-Ca^2+^	Polyacrylamide/Sodium alginate-CaCl_2_/LiBr
PS-Al^3+^	Polyacrylamide/Sodium alginate-AlCl_3_·6H_2_O/LiBr
PS-Zr^4+^	Polyacrylamide/Sodium alginate-ZrOCl_2_·8H_2_O/LiBr
