# Supplementary material for: A High-Strength, Anti-Swelling Sodium Alginate/Polyacrylamide Hydrogel Strain Sensor for Underwater Motion Monitoring and Information Transmission
Source: Gels. 2026 May 28;12(6):468. doi: 10.3390/gels12060468 (PMC13298718; doi:10.3390/gels12060468)
Supplement: Supplementary file 1 [file gels-12-00468-s001.zip › gels-4321424-supplementary.pdf]

Supplementary Materials

Article

A High-Strength, Anti-Swelling Sodium Alginate/Polyacrylamide Hydrogel Strain Sensor for Underwater Motion Monitoring and Information Transmission

Xuecui Song, Jing Guo *, Wei Chen, Mengya Liu, Yihang Zhang, Wenhui Xiao and Fucheng Guan

School of Textile and Material Engineering, Dalian Polytechnic University, Dalian 116034, China; songxuecui@163.com (X.S.); ivychen0310@163.com (W.C.); liumengya08@163.com (M.L.); 18263825182@163.com (Y.Z.); 15832395178@163.com (W.X.); gfc6322577@163.com (F.G.)

* Correspondence: guojing@dlpu.edu.cn

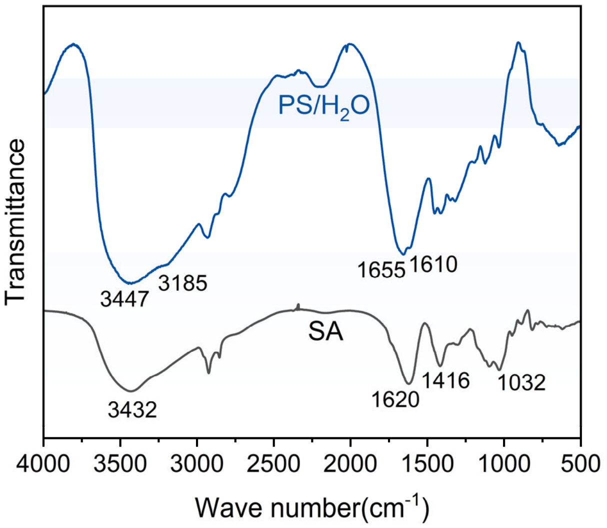

Figure S1. FTIR spectra of SA and PS/H_2O.

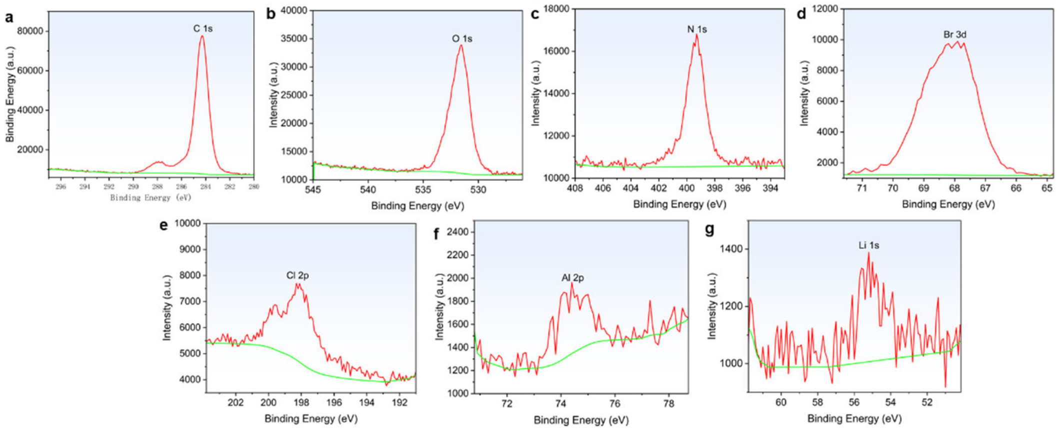

Figure S2. High-resolution XPS spectra of different elements in the PS-Al^{3+} hydrogel.

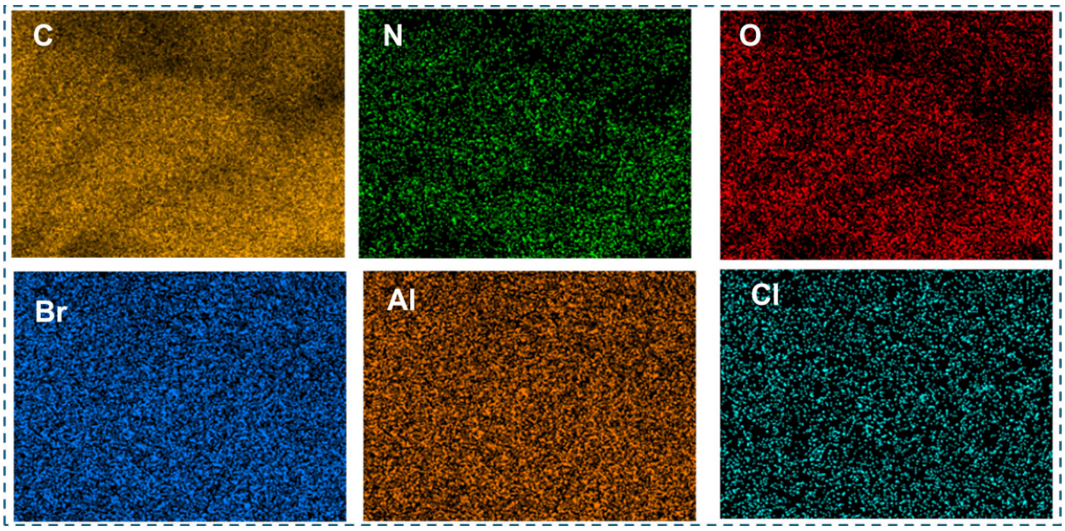

Figure S3. EDS mapping of the PS-Al^{3+} hydrogel.

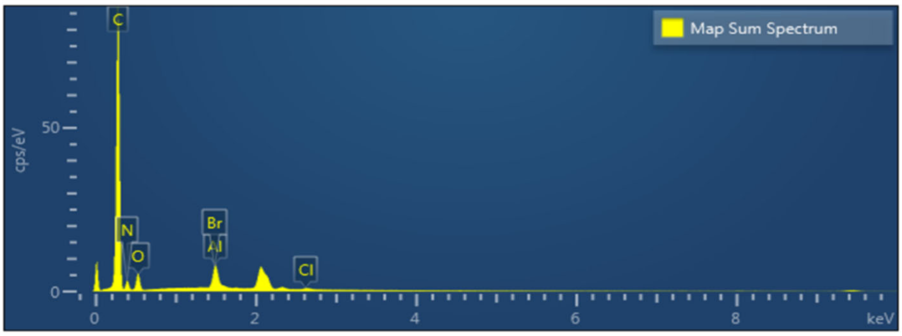

Figure S4. EDS spectra of the PS-Al^{3+} hydrogel.

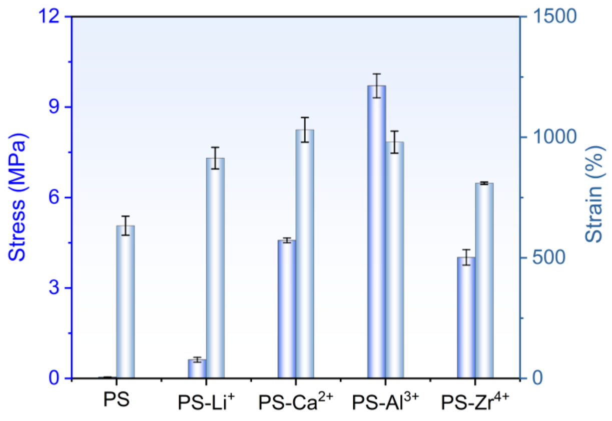

Figure S5. Tensile strength and elongation at break of five hydrogels.

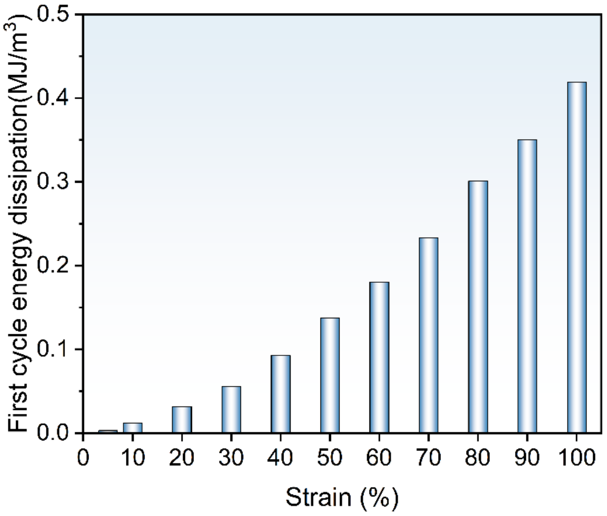

Figure S6. Hysteretic loss of the PS-Al^{3+} hydrogel during continuous loading and unloading cycles (with the maximum strain set at 5%–100%).

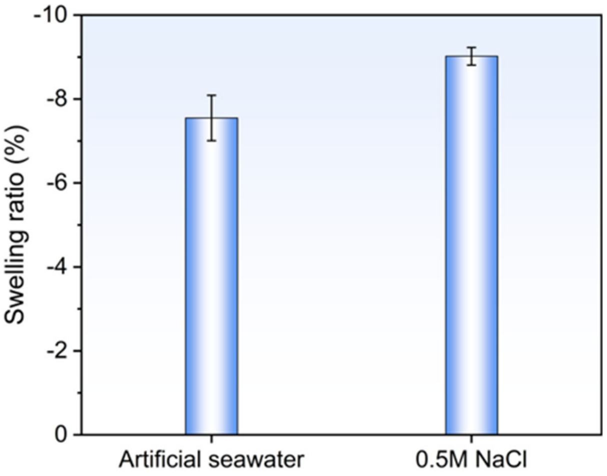

Figure S7 Swelling ratios of PS-Al^{3+} hydrogels in artificial seawater and 0.5 M NaCl solution.

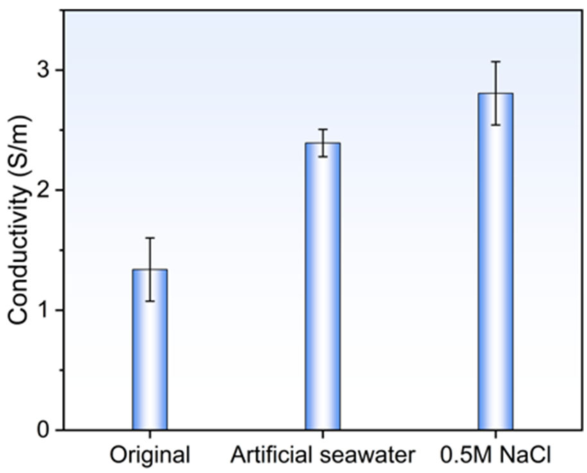

Figure S8 Conductivity of PS-Al^{3+} hydrogels before and after reaching swelling equilibrium in artificial seawater and 0.5 M NaCl solution.

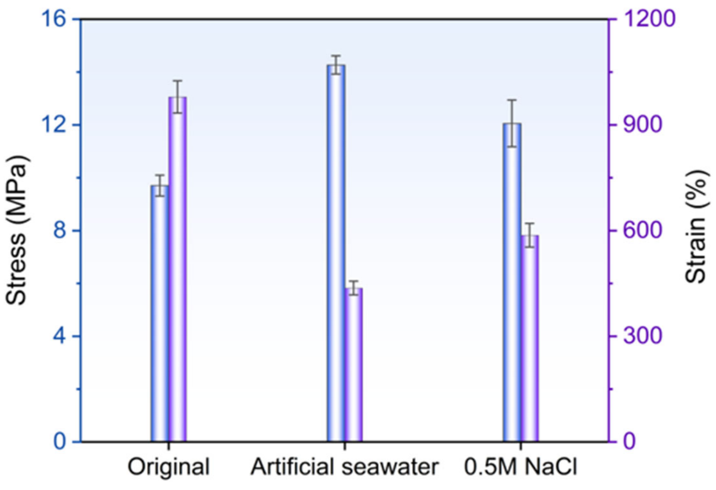

Figure S9 Tensile strength and elongation at break of PS-Al^{3+} hydrogels before and after reaching swelling equilibrium in artificial seawater and 0.5 M NaCl solution.

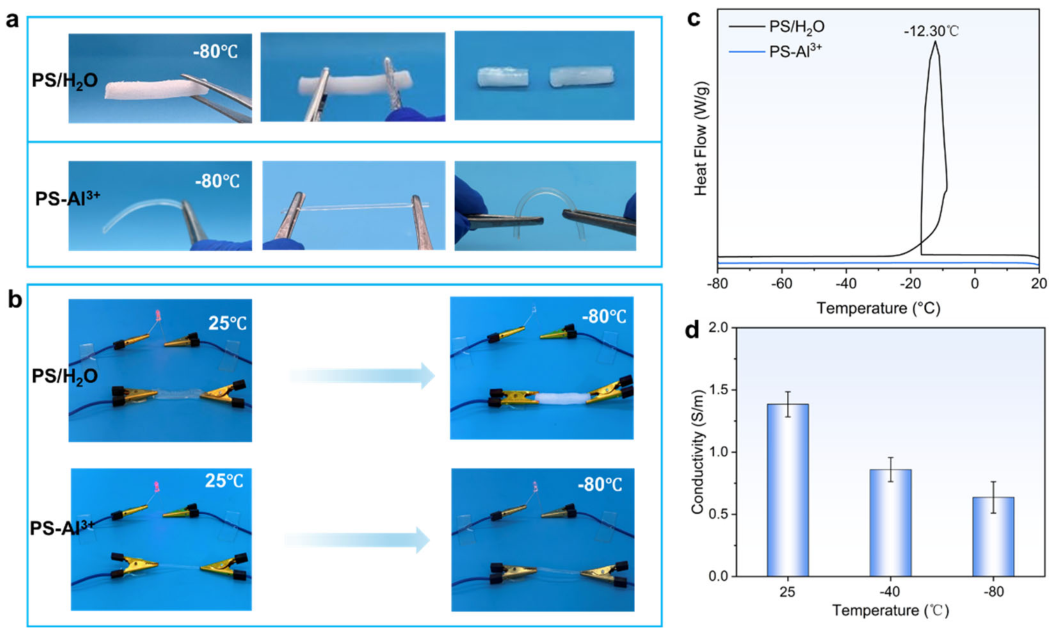

Figure S10. Freeze resistance of PS/H_2O and PS-Al^{3+} hydrogels. (a) Photographs showing the mechanical flexibility of both hydrogels at -80 °C. (b) Photographs demonstrating the electrical conductivity of both hydrogels at 25°C and -80 °C. (c) DSC curves of both hydrogels. (d) Electrical conductivity of PS-Al^{3+} hydrogel at different temperatures.
